# Axon-Specific Mitochondrial Pathology in SPG11 Alpha Motor Neurons

**DOI:** 10.3389/fnins.2021.680572

**Published:** 2021-07-07

**Authors:** Fabian Güner, Tatyana Pozner, Florian Krach, Iryna Prots, Sandra Loskarn, Ursula Schlötzer-Schrehardt, Jürgen Winkler, Beate Winner, Martin Regensburger

**Affiliations:** ^1^Department of Stem Cell Biology, Friedrich-Alexander-Universität Erlangen-Nürnberg, Erlangen, Germany; ^2^Department of Ophthalmology, Friedrich-Alexander-Universität Erlangen-Nürnberg, Erlangen, Germany; ^3^Department of Molecular Neurology, Friedrich-Alexander-Universität Erlangen-Nürnberg, Erlangen, Germany; ^4^Center for Rare Diseases Erlangen, University Hospital Erlangen, Erlangen, Germany

**Keywords:** SPG11, hereditary spastic paraplegia, alpha motor neuron, induced pluripotent stem cells, mitochondria, axonal transport

## Abstract

Pathogenic variants in *SPG11* are the most frequent cause of autosomal recessive complicated hereditary spastic paraplegia (HSP). In addition to spastic paraplegia caused by corticospinal degeneration, most patients are significantly affected by progressive weakness and muscle wasting due to alpha motor neuron (MN) degeneration. Mitochondria play a crucial role in neuronal health, and mitochondrial deficits were reported in other types of HSPs. To investigate whether mitochondrial pathology is present in SPG11, we differentiated MNs from induced pluripotent stem cells derived from SPG11 patients and controls. MN derived from human embryonic stem cells and an isogenic SPG11 knockout line were also included in the study. Morphological analysis of mitochondria in the MN soma versus neurites revealed specific alterations of mitochondrial morphology within SPG11 neurites, but not within the soma. In addition, impaired mitochondrial membrane potential was indicative of mitochondrial dysfunction. Moreover, we reveal neuritic aggregates further supporting neurite pathology in SPG11. Correspondingly, using a microfluidic-based MN culture system, we demonstrate that axonal mitochondrial transport was significantly impaired in SPG11. Overall, our data demonstrate that alterations in morphology, function, and transport of mitochondria are an important feature of axonal dysfunction in SPG11 MNs.

## Introduction

Hereditary spastic paraplegia (HSP) is a heterogeneous group of upper motor neuron (MN) diseases with the predominant clinical symptoms of progressive lower limb spasticity and weakness. The neuropathological correlate of HSP is degeneration of long corticospinal tract axons (Giudice et al., 2014; Blackstone, 2018; Parodi et al., 2018; List et al., 2019). HSPs can be classified as either pure or complicated (Harding, 1983). Complicated forms are defined by the presence of additional symptoms, including intellectual disability, thin corpus callosum, seizures, ataxia, and peripheral neuropathy (Harding, 1983; Giudice et al., 2014; Schüle et al., 2016; Pozner et al., 2020). More than 72 genes and loci underlying HSP have been identified to date.

Pathogenic variants in *SPG11* (the gene encoding spatacsin) are the most common cause of autosomal-recessive complicated HSP with thin corpus callosum (Stevanin et al., 2007). In addition to intellectual disability, motor neuropathy is commonly observed in SPG11 patients (Giudice et al., 2014; Pensato et al., 2014; Manole et al., 2016; Schüle et al., 2016; Faber et al., 2018a). While cognitive impairment and lower limb spasticity are early symptoms, motor neuropathy tends to emerge later in the disease course, albeit with significant progression and major impact on the patients’ disability (Manole et al., 2016). Phenotypically, pathogenic variants in *SPG11* may also present as autosomal recessive juvenile amyotrophic lateral sclerosis (ALS5) or as a pure motor neuropathy (Orlacchio et al., 2010; Denora et al., 2016), indicating that motor neuropathy is a common feature of different phenotypic presentations of pathogenic *SPG11* variants. Moreover, degeneration of alpha MNs was observed in two different animal models of *SPG11*. In the zebrafish model, depletion of spatacsin impaired MN development (Martin et al., 2012). Degeneration of cortical neurons and MN was also present in a *Spg11* knockout mouse model (Branchu et al., 2017).

A recent study revealed impaired mitochondrial morphology and function in a neuronal model of SPG15: mitochondrial length, aspect ratio, and axonal density were significantly decreased (Denton et al., 2018). As multiple lines of evidence indicate overlapping mechanisms in SPG11 and SPG15 (Hirst et al., 2013; Chang et al., 2014; Pensato et al., 2014; Varga et al., 2015), we aimed to study mitochondria in SPG11. Previously, *SPG11* loss of function has been linked to impaired autophagic lysosome reformation in primary patients’ fibroblasts, in HeLa cells and in the *SPG11* knockout mouse model (Chang et al., 2014; Branchu et al., 2017; Vantaggiato et al., 2019). Moreover, this dysfunctional lysosome reformation led to accumulation of cholesterol and gangliosides along with altered cytosolic calcium homeostasis (Boutry et al., 2018, 2019). Data on mitochondria in SPG11, however, are lacking to date.

We hypothesized that morphology, function, and transport of mitochondria are impaired in SPG11. In a new model of MN derived from SPG11 patients’ induced pluripotent stem cells (iPSCs) and from gene edited human embryonic stem cells, we demonstrate altered mitochondrial morphology and mitochondrial polarization, along with an accumulation of neuritic aggregates in SPG11. Furthermore, disruption of axonal mitochondrial transport suggests an involvement of mitochondria in SPG11 neuritic dysfunction.

## Materials and Methods

### Patients, Fibroblast Derivation, and Reprogramming of iPSC

For this study, two patients with compound heterozygous pathogenic variants in the SPG11 gene and three age and gender matched healthy controls were included (Table 1). The controls were not related to both patients, and all study participants were of Caucasian background. The variants of both SPG11 patients cause biallelic premature stop codons within the *SPG11* transcript, leading to a loss of function of spatacsin, the protein encoded by *SPG11* (Hehr et al., 2007; Stevanin et al., 2007). We have previously shown that forebrain iPSC-derived models of both patients have deficits in neuronal progenitor cell proliferation, forebrain organoid growth, axonal outgrowth, and axonal transport (Pérez-Brangulí et al., 2014, 2019; Mishra et al., 2016). However, no models of the alpha MN have been generated or analyzed so far. Both patients exhibited severe motor neuropathy as shown by pronounced distal atrophy of upper and lower limbs, nearly absent potentials in motor nerve conduction studies and a Charcot Marie Tooth neuropathy score of 19 (Table 1; Hehr et al., 2007). Human fibroblasts were obtained from dermal punch biopsies of the upper arm complying with the local Institutional Review Board approval (No. 4120: “Generierung von humanen neuronalen Modellen bei neurodegenerativen Erkrankungen” and No. 259_17B: “Biobank zur Untersuchung von Biomarkern und Generierung humaner Zellmodelle bei Neurologischen Erkrankungen”) and respective written informed consents. The generation of iPSC was conducted as described previously (Pérez-Brangulí et al., 2014; Popp et al., 2018). In brief, iPSC were reprogrammed using non-integrating viral transduction of the transcription factors OCT3/4, c-MYC, SOX2, and KLF4 (Sendai reprogramming kit, ThermoFisher). IPSCs were maintained in StemMACS iPS-Brew (Milteny Biotech) on Geltrex-coated plates. Pluripotency of iPSC lines was confirmed by FACS-based analysis of Tra1-60 expressing cells, which was at least 90% positive for each line (Pérez-Brangulí et al., 2014; Popp et al., 2018). In addition, all lines were screened for stable karyotype using the G-banding chromosomal analysis and analysis of copy number variations >100 kb (Popp et al., 2018). Per individual, two cell lines were established. The specific clones and lines that were employed for each experiment are indicated in the respective Supplementary Figures.

**TABLE 1:** Clinical phenotypes and genetic characteristics of SPG11 patients and controls.

	**SPG11-1**	**SPG11-2**	**Control-1**	**Control-2**	**Control-3**
SPG11 mutations	exon16: c.3036C > A exon30: c.5798delC	exon2: c.267G > A intron6: c.1457-2A > G	–	–	–
Protein change	Tyr1012X/Ala1933fs1951X	Trp89X/Glu486fs508X			
Sex	Female	Female	Female	Female	Female
Age at onset/age at examination (years)	20/40	31/50	–/45	–/66	–/28
SPRS (0–52)	37	36	0	0	0
FDS (0–8)	7	7	0	0	0
CMTNS (0–36)	19	19	0	0	0
CMAP UL (mV)	0	0.3	n/a	n/a	n/a
CMAP LL (mV)	0	0	n/a	n/a	n/a
EMG hand muscle	Acute and chronic denervation	Acute and chronic denervation	n/a	n/a	n/a
Thenar atrophy	+++	+++	–	–	–
Hypothenar atrophy	+++	++	–	–	–
Claw hand	++	++	–	–	–
Lower limb atrophy	++	++	–	–	–
Fasciculations	+	+	–	–	–
Cognitive impairment	+	+	–	–	–
Wheelchair dependency	+	+	–	–	–
MRI abnormalities	Cortical atrophy, WML, TCC	Cortical atrophy, WML, TCC	–	–	–
Age at sampling of fibroblasts	34	43	45	65	28
iPSC clones	SPG11-11, SPG11-12	SPG11-21, SPG11-22	Control-11, Control-12	Control-21, Control-22	Control-31, Control-32
Full identifier of individual	UKERi4AA-S	UKERiK22-S	UKERi33Q-S	UKERi82A-S	UKERi55O-S

*SPRS, spastic paraplegia rating scale; FDS, functional disability scale; CMTNS, Charcot Marie Tooth Neuropathy Score; CMAP, compound muscle action potential; UL, upper limb (abductor pollicis brevis muscle); LL, lower limb (abductor hallucis brevis muscle); EMG, electromyography obtained from intrinsic hand muscles; TCC, thin corpus callosum; WML, white matter lesions; iPSC, induced pluripotent stem cells.*

### Human Embryonic Stem Cell Model

An *SPG11* knockout human embryonic stem cell line derived from the HUES6 line (SPG11-HUES6) and its isogenic control line (wt-HUES6 which were subjected to similar culture conditions of the genome editing process as SPG11-HUES6 but were not genetically modified) were used as additional cell lines. The generation of this clonal *SPG11* knockout line by CRISPR/Cas9 mediated gene editing including its characterization and the absence of off-target events were published previously (Pozner et al., 2018). All experiments with HUES6 cells and the derived neurons were conducted in accordance with the German Stem Cell Act (RKI, “63. Genehmigung” to BW).

### Motor Neuron Differentiation

For differentiation of iPSC into MNs, we adapted previous protocols of MN differentiation (Chambers et al., 2009; Du et al., 2015; Maury et al., 2015; Krach et al., 2018). In brief, iPSCs were differentiated in a monolayer, using the differentiation medium DMEM-F12 containing 1% N-2, 2% B-27 supplements (Thermo Fisher Scientific) and ascorbic acid (50 μM). For 15 days, dual SMAD inhibition was performed with LDN-193189 (0.1 μM) and SB431542 (10 μM). CHIR99021 (4 μM) was added from day 1 through 6. From day 7 through 22, purmorphamine (600 nM) and retinoic acid (1.5 μM) were added. During the first 15 days, the medium was changed daily, afterward the medium was changed every other day. On day 15, cells were dissociated and seeded for final differentiation until day 30, at a density of 50,000 cells per 1.9 cm^2^. From day 15 on, growth factors BDNF, GDNF, and CNTF (all 2 ng/ml) were added. DAPT (2 μM) was added on days 23 through 25. In order to prevent cell detachment of the high density cultures, culture plates were coated with Matrigel (ThermoFisher) until day 15 and Poly-D-Lysine/Laminin (Sigma) during differentiation from day 15 on. For a low-threshold quantification of MNs, a lentivirus overexpressing GFP under control of the Hb9 promoter was added to the culture on day 30 of differentiation (Marchetto et al., 2008) and the number of positive cells over DAPI was quantified 2 days later (Supplementary Figure 1D).

### Quantitative Real-Time PCR

RNA was extracted using RNeasy kit (Qiagen) according to the manufacturer’s instructions. A total of 500 ng RNA were reverse-transcribed into cDNA in 20 μl reaction solution by QuantiTect Reverse Transcription Kit (Qiagen). One microliter of cDNA was used for real-time polymerase chain reaction (qPCR). The qPCR program was as follows: 95°C 10 min/40 cycles of 95°C for 15 s and 60°C for 1 min/95°C for 15 s/60°C for 30 s/95°C for 15 s. The following primers were used: *GAPDH* TGTTGCCATCAATGACCCCTT and CTCCACGACGTACTCAGCG, *HPRT* CCTGGCGTCGTGA TTAGTG and TCCCATCTCCTTCATCACATC, *OCT4* GTGTTCAGCCAAAAGACCATCT and GGCCTGCATGAG GGTTTCT, *PAX6* TCTTTGCTTGGGAAATCCG and CTGCCCGTTCAACATCCTTAG, *OLIG2* GGGCCACAAGTTA GTTGGAA and GAGGAACGGCCACAGTTCTA, *ISL1* TGTTTGAAATGTGCGGAGTG and GCATTTGATCCCGT ACAACC, *CHAT* CAGCCCTGCCGTGATCTTT and TGTAGCTGAGTACACCAGAGATG, *HB9* GCACCAGTTCA AGCTCAACA and TTTGCTGCGTTTCCATTTC. Samples were analyzed in duplicates. Expression levels were compared using the 2^–ΔΔCT^ method, using *GAPDH* and *HPRT* as housekeeping genes.

### Analysis of Cellular Proliferation

For the analysis of proliferation at the progenitor cell stage, MN progenitors were treated with BrdU (30 μM; Sigma) for one hour on day 21 of differentiation and subsequently fixed for staining with BrdU and DAPI.

### Analysis of Neurite Growth

Single neurons within regular 24-well cultures were labeled using a low titered transfection with the pEF1-dTomato plasmid as described previously (Pozner et al., 2018). In brief, neurons were transfected on day 30 of differentiation using Lipofectamine, 2000 (Invitrogen, reagent:DNA ratio 2:1) and fixation was performed 48 h after transfection. The cells were stained for the expression of Hb9 and DAPI (see below) and were mounted on slides. DTomato/Hb9 double positive neurons that did not show substantial overlap with neighboring cells were recorded on the Observer.Z1 fluorescence microscope (Zeiss). Semi-automated tracing was performed using the NeuronJ plugin of ImageJ/Fiji (Schindelin et al., 2012).

### Mitochondrial and Lysosomal Labeling and Treatment

Mitochondria were stained with MitoTracker^®^ Red CMXRos (M7512 ThermoFisher, MT-R). Acidic organelles were stained by Lysotracker Green DND-26 (#8783 Cellsignal, LT-G). For Mitotracker and Lysotracker stainings, cells were incubated for 30 min with Mitotracker (50 nM) and Lysotracker (50 nM) in prewarmed, freshly prepared culture medium (50% total medium change). In MN grown in microfluidic chambers, mitochondria were labeled by addition of a lentivirus overexpressing MitoDsRed (Prots et al., 2018). To analyze mitochondrial membrane potential, JC-1 (T3168, Invitrogen) was added to the medium at a final concentration of 1 μg/ml (50% medium change to prevent detachment) and cells were recorded after 60 min. As a positive control of mitochondrial depolarization, CCCP (50 μM final concentration, diluted in ethanol) was added to the medium 30 min before imaging. Lysosomes were labeled using immunohistochemistry for Lamp1 (see below) on day 31 differentiated neuronal cultures. In the ImageJ/Fiji software, the Lamp1 channel was thresholded using the auto function and the area of Lamp1 was determined within the region of the cell soma. For the quantification of Lamp1 positive organelles (LPO), enlarged perinuclear Lamp1 organelles were manually counted on blindcoded slides.

### Microfluidic Chamber Cultures

**FIGURE 1:**
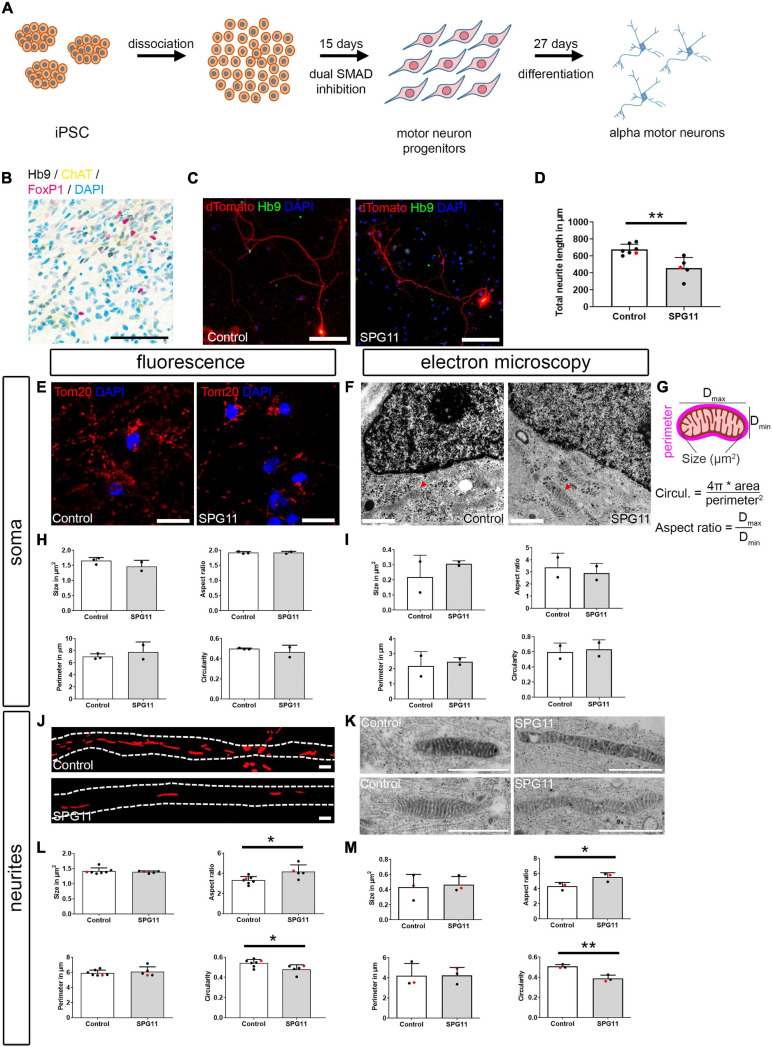
Characterization of iPSC-derived alpha motor neurons (MNs) and compartment-specific changes of mitochondrial morphology in SPG11. **(A)** Paradigm of differentiation by small molecule dual SMAD inhibition. **(B)** Immunohistochemistry of d27 mature MNs (belonging to line Control-11) shows an expression of the alpha MN markers Hb9, ChAT, and FoxP1. Channels are shown in CMYK colors to clarify colocalization. **(C)** Single MN were labeled by transfection with pEF1a:dTomato plasmid and counterstained with Hb9 to confirm MN identity. **(D)** Quantification of total neuritic length of dTomato labeled MN (*n* = 7 control and 5 SPG11 lines; HUES6 lines marked red). **(E)** Representative images of mitochondria within the MN soma, as labeled by Tom20 in Control and SPG11. **(F)** Representative electron micrographs of mitochondria (red arrowheads) within the soma of Control and SPG11. **(G)** Schematic representation and calculation of mitochondrial morphology parameters. **(H,I)** Quantification of mitochondrial morphology parameters size, aspect ratio, perimeter, and circularity upon fluorescence labeling (**H**; *n* = 3 control and 2 SPG11 lines) and ultrastructural analysis (**I**; *n* = 2 control and 2 SPG11 lines). **(J)** Representative micrographs of mitochondria within the MN neurites in Control and SPG11. Dashed lines indicate neurite outlines. **(K)** Representative electron micrographs of mitochondria within the neurites of Control and SPG11. **(L,M)** Quantification of neuritic mitochondrial morphology parameters size, aspect ratio, perimeter, and circularity upon fluorescence labeling (**L**; *n* = 7 control and 5 SPG11 lines; HUES6 lines marked red) and ultrastructural analysis (**M**; *n* = 3 control and 3 SPG11 lines; HUES6 lines marked red). Scale bars: **(B,C)** 100 μm; **(E)** 10 μm; **(F)** 1 μm; **(J)** 1 μm; **(K)** 1 μm. *P < 0.05, **P < 0.01.

**FIGURE 2:**
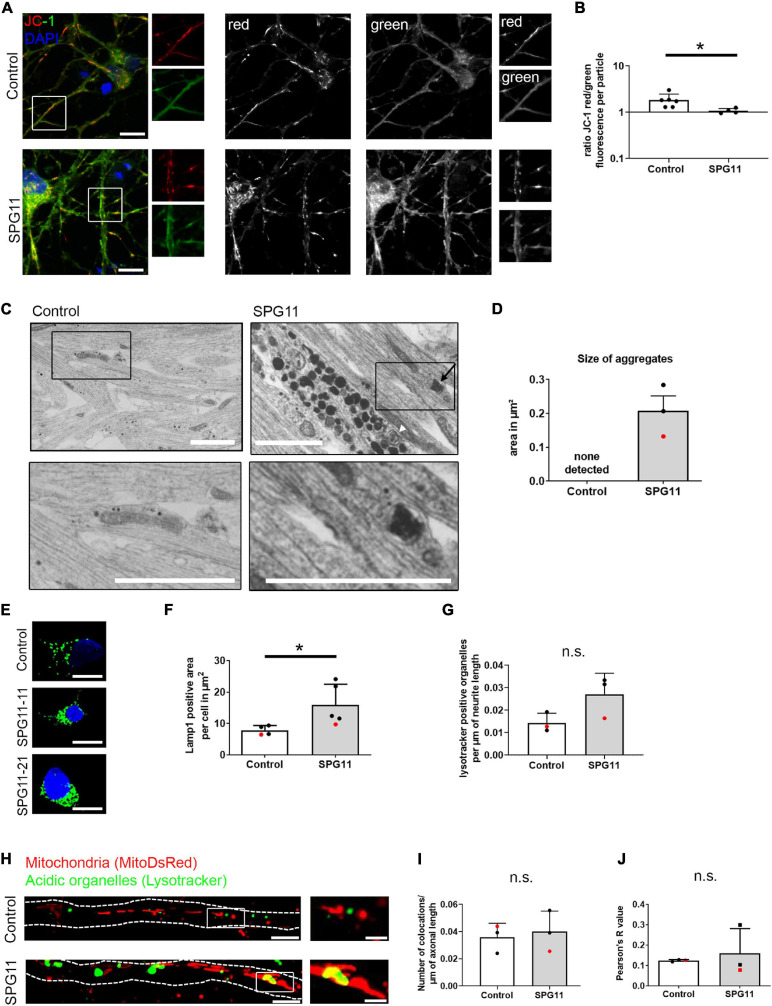
Mitochondrial function and neuritic aggregates in SPG11. **(A)** Analysis of membrane potential using the JC-1 dye. Single color channel magnified areas from the white boxes are shown on the right side of each panel. **(B)** Quantification of the mean fluorescence ratio red/green per mitochondrion (*n* = 6 control and 4 SPG11 lines). **(C)** Neurites of mature MNs were analyzed by electron microscopy. SPG11 neurites exhibited electron-dense aggregates, including double membrane engulfed organelles (black arrow) and multivesicular bodies (white arrowhead). These aggregates were not observed in control MN. Black boxes are shown magnified in the lower line. **(D)** Quantification of the area of the observed electron-dense neuritic aggregates in SPG11 (*n* = 3 control and 3 SPG11 lines; HUES6 lines marked red). **(E)** MN were labeled with immunohistochemistry for Lamp1. **(F)** Quantification of Lamp1 positive area per MN soma (*n* = 4 control and 5 SPG11 lines; HUES6 lines marked red). **(G)** Quantification of the number of lysotracker positive large organelles per μm of neurite length in Control and SPG11 (*n* = 3 control and 3 SPG11 lines; HUES6 lines marked red). **(H)** MNs were labeled by expression of MitoDsRed and by staining with Lysotracker Green. Dotted lines indicate axon outlines. White boxes within images on the left are shown magnified on the right. **(I)** Mitochondria and Lysotracker double-positive particles were counted manually and blinded to genotype, in relation to axonal length (*n* = 3 control and 3 SPG11 lines; HUES6 lines marked red). **(J)** Pearson’s R-value of correlation analyzing overlap of mitochondria and lysotracker-positive organelles (*n* = 3 control and 3 SPG11 lines; HUES6 lines marked red). Scale bars: **(A)** 10 μm; **(C)** 1 μm; **(E)** 10 μm; **(H)** 10 μm/2.5 μm. *P < 0.05.

**FIGURE 3:**
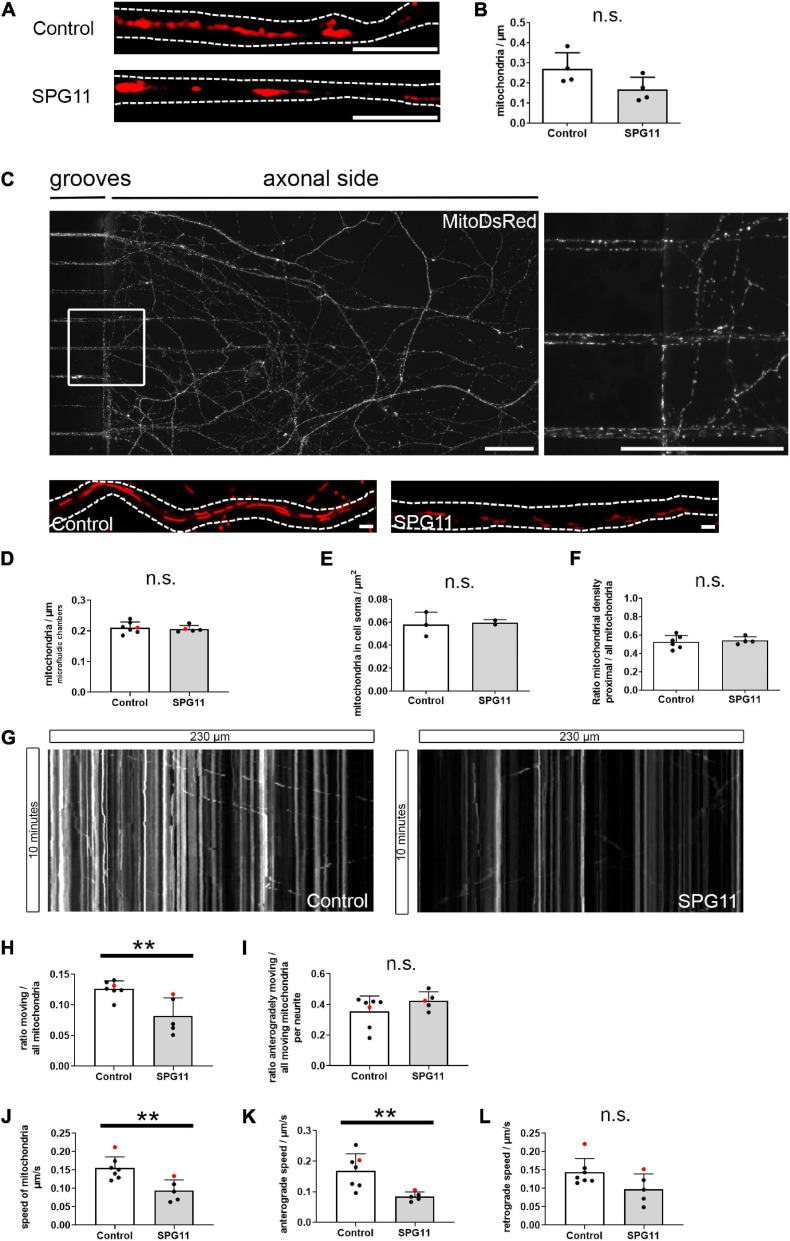
Mitochondrial density and mitochondrial axonal transport in SPG11. **(A)** Neurites of MNs were stained with Mitotracker. Dotted lines indicate neurite outlines. **(B)** Mitochondrial density was determined upon quantification of mitochondria related to neuritic length (*n* = 4 control and 4 SPG11 lines). **(C)** Representative micrograph of MNs grown in microfluidic chambers, extending from grooves into the axonal side. Magnification of the boxed area is shown on the right side. **(D)** Quantification of the density of mitochondria within the axonal grooves of microfluidic chambers (*n* = 7 control and 5 SPG11 lines; HUES6 lines marked red). **(E)** Quantification of the density of mitochondria within the soma of MN cultures (*n* = 3 control and 2 SPG11 lines). **(F)** Quantification of the ratio of the number of mitochondria located within the proximal half of axons over the total number of mitochondria (*n* = 6 control and 4 SPG11 lines). **(G)** Sample kymographs of mitochondria in grooves of microfluidic chambers over 10 min. **(H–L)** Quantification of axonal mitochondrial transport (*n* = 7 control and 5 SPG11 lines for all analyses; HUES6 lines marked red). **(H)** Quantification of the ratio of moving mitochondria over total mitochondria per axon. **(I)** Quantification of the ratio of anterogradely moving mitochondria over all moving mitochondria per axon. **(J)** Combined analysis of the speed of all moving mitochondria in μm/s. **(K)** Subanalysis of the speed of anterogradely moving axonal mitochondria in μm/s. **(L)** Subanalysis of the speed of retrogradely moving axonal mitochondria in μm/s. ** *P* < 0.01, *n.s.* no significant change. Scale bars: **(A)** 5 μm; **(C)** 50 μm (upper images), 1 μm (lower images).

Neurons were cultured in microfluidic chambers (SND450, Xona Microfluidics) as described previously (Prots et al., 2018). In brief, 80,000 MN progenitors and 10,000 astrocytes (primary human cerebellar astrocytes, HA-c, ScienCell Research Laboratories) were seeded on the soma side, and 20,000 astrocytes on the axonal side. Cells located in the soma side were lentivirally infected 2 days post-seeding at a multiplicity of infection of 1, with an incubation time of 48 h, followed by a complete medium change. Mito-DsRed-positive mitochondria were recorded on day 30 after differentiation when the axons had projected through the 450 μm-long microgrooves reaching the axonal side. Microfluidic chambers were used to analyze morphology of neuritic mitochondria (Figure 1), colocalization of Lysotracker with mitochondria (Figure 2), axonal density of mitochondria (Figure 3), and mitochondrial axonal transport (Figure 3).

### Analysis of Axonal Transport

Axonal transport was analyzed as described before (Prots et al., 2018). Specifically, on day 15 after induction of differentiation (day 30 from iPSC), live-cell imaging of the grooves of the microfluidic chambers was performed using a fluorescence microscope Axio Observer.Z1 (Carl Zeiss) at a 63× objective using oil immersion, a frame rate of 0.2/s and autofocus. All recordings were performed in the proximal part of the grooves. At least 14 neurites were analyzed per line. Each recording lasted for 10 min. After acquisition, stage position correction was calculated in the Zen software. Videos were opened in ImageJ/Fiji and corrected for bleaching with the integrated program function. Axons were traced with the multi-point selection tool at a line width of five pixels. Kymographs were created with the “Multiple Kymograph” function. For each kymograph, density of total mitochondria was determined and moving mitochondria were manually identified. To qualify as a moving particle, it had to be displaced at least 3 μm between the start and end of the recording. For the calculation of transport speed, the mean speed covering the complete recording time of 10 min was calculated. Moreover, the direction of displacement was recorded.

### Immunofluorescence Staining and Microscopy

Immunocytochemical and ultrastructural analyses were performed on MNs grown on coverslips in 24-well plates, coated with Poly-D-Lysine/Laminin. Cells were washed with phosphate buffered solution (PBS) once, then fixed with 4% paraformaldehyde (PFA) in PBS for 15 min at room temperature and again washed with PBS three times. For immunocytochemistry, cells were blocked and permeabilized with 0.1% Triton X-100 and 3% Donkey Serum (DS) in PBS for 30 min at room temperature. Primary antibodies were diluted in 3% DS in PBS and incubated overnight at 4°C. After washing twice with PBS, secondary antibodies (1:500, Alexa) were incubated for 1 h at room temperature in the dark. DAPI (1:5,000 or 1:10,000 in PBS) was incubated for 1 min in the dark. Cells were subsequently washed with PBS three times. MN progenitors were stained with BrdU (1:100, rat, Abcam; with an additional preceding DNA denaturation step) or Sox2 (1:300, rabbit, Cell Signaling). MN were stained with Hb9 (1:50, mouse IgG1k, 81.5C10, DSHB Iowa), choline acetyltransferase (ChAT; 1:300, goat IgG, AB144P, Millipore), FoxP1 (1:5,000, rabbit, ab16645, Abcam) and Tom20 (1:250, rabbit, sc-11415, Santa Cruz). For lysosome detection, saponin was added at 0.5% in the blocking step, followed by incubation with Lamp1 (1:200, mouse monoclonal H4A3, ab25630, Abcam). Coverslips were mounted on glass object slides using Aqua Polymount (Polysciences Inc.) and dried overnight at 4°C. Images were acquired using a fluorescence microscope Axio Observer.Z1 (Carl Zeiss) at a 63× objective using oil immersion and Apotome. Imaging of JC-1 labeled MN was performed at a 20× objective.

### Mitochondrial Quantification

On day 30 from MN differentiation (day 45 after iPSC stage), neurites stained for mitochondria were chosen randomly. The ImageJ Mito-Morphology Macro (Dagda et al., 2009) was applied to analyze the number, the total area, the percentage area, the average size, the perimeter, the aspect ratio and circularity of the mitochondria found in SPG11 compared to control lines. Exposure times were set automatically for all object slides and the threshold-plugin was used to determine positive structures. To compare the density of mitochondria within the proximal versus distal part of the microfluidic chamber grooves, the ratio of proximal mitochondria over all mitochondria was calculated.

Membrane potential analyses were performed on the level of single mitochondria using ImageJ. After automatic outlining of mitochondria in the green channel by thresholding, the mean intensity of both fluorophores was recorded for each particle. The ratio of red over green was calculated for each particle.

### Colocalization

Colocalization analysis of MitoDsRed and Lysotracker-Green for dense particles was performed within the grooves of XONA microfluidic chambers using the automatized colocalization threshold plugin Coloc2 for Fiji^1^. To minimize false positive detection, the red fluorescent area (i.e., mitochondria) within the axon was defined as the region of interest, and fluorescence detection filters were set to minimize cross-bleaching. Due to its superiority in automatic colocalization analysis compared to the Manders coefficient of colocalization, we calculated the Pearson’s r coefficient (Adler and Parmryd, 2010). The manual colocalization analysis was performed by manual counting of single-positive and double-positive mitochondria. Manually counted colocalizations are presented as the number of colocalized particles per linear axonal length.

### Electron Microscopy

Transmission electron microscopy was performed as previously described (Schlötzer-Schrehardt et al., 2012). In brief, MN cultures were grown on plastic coverslips in 24-multiwell plates, coated with Matrigel, with a total cell number of 100,000 per well. MN were fixed at day 20 of differentiation (day 35 from iPSC) in 2.5% glutaraldehyde in 0.1 M phosphate buffer, post-fixed in 2% buffered osmium tetroxide, dehydrated in graded alcohol concentrations and embedded in epoxy resin according to standard protocols. Ultrathin horizontal sections were stained with uranyl acetate and lead citrate and were examined with a transmission electron microscope (EM 906E; Carl Zeiss NTS). Mitochondria of the soma were included in morphological analyses when the respective nucleus was visible on the same slide. The analysis of neuritic mitochondrial morphology was performed within the neuritic compartment, i.e., on images that showed no neuronal soma. Neurites were only analyzed if they extended throughout the image to minimize off-plane measurements (i.e., on neurites crossing the section transversely). Mitochondria were outlined manually in ImageJ. Size in μm^2^, aspect ratio (i.e., the ratio of maximum over minimum diameter) perimeter in μm and circularity (4π × area/perimeter^2^) were calculated (Figure 1G).

### Statistics

Counting analyses for Lysotracker-positive organelles, LPO, mitochondrial density, and axonal transport analyses were performed blinded for genotype. All data in the main figures are shown as mean ± SD. Statistical analyses were performed using Graph Pad Prism (GraphPad Software, La Jolla, CA, United States). For comparison of two groups, an unpaired, two-sided *t*-test was applied. Statistical significance was indicated by ^∗^ for *P* < 0.05 and ^∗∗^ for *P* < 0.01.

## Results

### Spinal MNs Generated From Fibroblasts as a Model of Motor Neuropathy

Since motor neuropathy is a disabling symptom found in a substantial proportion of SPG11 patients, we reprogrammed fibroblasts derived from two SPG11 patients and three healthy controls into iPSC. Motor neuropathy was severe in both patients. They showed marked atrophy of hands, distal arm, and leg muscles, along with paresis and electromyographic signs of acute and chronic denervation (Table 1; Hehr et al., 2007). In addition, HUES6 embryonic stem cells and CRISPR/Cas9 mediated *SPG11* knockout HUES6 were included.

Pluripotent stem cells were differentiated into MNs using a dual SMAD inhibition-based monolayer protocol and subsequent treatment with neurotrophic and caudalizing factors (Figure 1A). The MN identity was confirmed by quantitative real-time PCR comparing iPSC (day 0), MN progenitors (day 20), and mature MNs (day 27; Supplementary Figure 1A). At days 20 and 27, the pluripotency marker Oct4 was downregulated. On day 20, the neurogenic transcription factor Pax6 and the oligodendrocyte/MN transcription factor Olig2 were upregulated, with a subsequent decrease on day 27. The MN markers Islet1, ChAT, and Hb9 were upregulated both on days 20 and 27. The BrdU incorporation assay revealed that at day 21, the lines exhibited comparable proliferation of neural progenitor cells (Supplementary Figure 1B). The ratio of Sox2-positive neural progenitor cells also did not significantly differ between controls and SPG11 (Supplementary Figure 1C). In order to confirm MN identity, expression of ChAT and Hb9 was shown by immunocytochemistry (Figure 1B) and by a lentivirus expressing GFP under control of the Hb9 promoter (Supplementary Figure 1D).

### Neurite-Specific Morphological Changes in SPG11 Motor Neurons

We have previously shown that neurite length is decreased in corticospinal MNs derived from SPG11 iPSCs and from SPG11-HUES6 (Pérez-Brangulí et al., 2014; Pozner et al., 2018). To investigate whether neurite outgrowth is also impaired in SPG11 MN, neuritic tree morphology was visualized by overexpression of dTomato on day 30 of differentiation. There was a significant reduction of total neuritic length in SPG11 MN when compared to control lines (Figure 1D and Supplementary Figure 1F).

Since morphological changes in mitochondria reflect their dysfunction and since reduced length of mitochondria was previously reported in other types of HSP (Denton et al., 2018), we next investigated whether alterations in mitochondrial morphology were also present in SPG11. Using immunofluorescence and electron microscopy, we analyzed mitochondrial morphology in mature MNs from SPG11 patients and controls, and we separated between analyses of soma (Figures 1E–I) and neuritic processes (Figures 1J–M). For each condition, size, aspect ratio (i.e., the ratio of maximum and minimum diameter of mitochondria), perimeter, and circularity of mitochondria were determined (Figure 1G). Immunofluorescence analysis of Tom20 labeled mitochondria localized within the soma compartment revealed no significant changes between SPG11 and controls (Figure 1H and Supplementary Figure 1G). Because of its superior resolution, mitochondrial morphology was also investigated on electron microscopy recordings of MNs cultured in 24-multiwell plates. The analysis of mitochondria localized within the soma confirmed unchanged mitochondrial morphology in SPG11 (Figure 1I and Supplementary Figure 1H). Next, we analyzed mitochondrial morphology in the neurites of MNs (Figures 1J–M). Immunofluorescence analysis of SPG11 neurites revealed a significant increase in aspect ratio and a significant decrease in circularity of SPG11 neuritic mitochondria (Figure 1L and Supplementary Figure 1I). This was confirmed on ultrastructural analysis which showed a significant (31%) increase in aspect ratio and a significant (21%) decrease in circularity in SPG11 (Figure 1M and Supplementary Figure 1J). Overall, in SPG11 MN, neurites are shorter and mitochondria have a longer and thinner morphology.

### Reduced Mitochondrial Membrane Potential in SPG11 Motor Neurons

In order to test whether morphological changes were accompanied by functional impairment of mitochondria, we analyzed the membrane potential of MN cultures using the fluorescent probe JC-1 (Figure 2A). In polarized mitochondria, red and green fluorescence is emitted, whereas upon depolarization, predominantly red fluorescence is decreased. CCCP depolarized mitochondria served as a positive control for both groups (Supplementary Figures 2A,B). Calculating the ratio of red over green fluorescence per mitochondrion, a significantly decreased membrane potential was observed in SPG11 (Figure 2B). These data show that mitochondrial membrane potential is reduced in SPG11 MNs.

### Ultrastructural Evidence of Neuritic Aggregates in SPG11 MN

Previous studies reported an accumulation of autophagosomes in SPG11, due to a deficit in autophagolysosome reformation (Renvoisé et al., 2014; Branchu et al., 2017; Vantaggiato et al., 2019; Pozner et al., 2020; Khundadze et al., 2021). Ultrastructural analysis of SPG11 MNs confirmed this finding, revealing presence of neuritic electron-dense aggregates in SPG11, but not in control MN neurites which exhibited small electron dense lipofuscin particles only (Figures 2C,D and Supplementary Figure 2C). While density measurements were not performed due to the restricted size of the fields of view and while the number of surrounding membranes and their content could not be further determined for most aggregates, some of them contained multiple membranes resembling multivesicular bodies and were present in proximity to mitochondria (Figure 2C).

### Increased Number of Lysosomes in SPG11 MNs

The common hallmark of SPG11 loss of function models is the accumulation of lysosomes (Pérez-Brangulí et al., 2014; Renvoisé et al., 2014; Varga et al., 2015; Branchu et al., 2017; Pozner et al., 2018). To determine whether lysosomal alterations were also present in SPG11 MNs, we performed immunohistochemistry for Lamp1 (Figure 2E). Analyzing Lamp1 positive area per MN (soma compartment), there was a significant increase of Lamp1 in SPG11 compared to controls (Figure 2F and Supplementary Figure 2D). We also assessed the density of lysotracker positive organelles in the neuritic compartment. There were no significant changes although there was a trend toward an increase of acidic organelles in SPG11 neurites (Figure 2G and Supplementary Figure 2E).

We next analyzed the colocalization of mitochondria and acidic organelles in MNs expressing MitoDsRed and stained with lysotracker (Figure 2H and Supplementary Figure 2F). Colocalization of mitochondria and lysotracker positive organelles per axonal length were manually counted. There was no significant change in colocalized organelles per μm of neuritic length in SPG11 compared to controls (Figure 2I and Supplementary Figure 2G). An additional colocalization analysis also showed no significant change in Pearson’s R value of colocalization (Figure 2J and Supplementary Figure 2H). In summary, these data confirm that lysosomes accumulate also in SPG11 MNs, but there was no specific alteration in lysosomal clearance of mitochondria.

### Impaired Transport of Mitochondria in SPG11 MN

To investigate whether altered morphology and membrane potential of mitochondria were associated with an altered distribution of mitochondria within MN neurites, we first quantified their density within regular coverslip cultures stained with mitotracker (Figure 3A). We observed no significant change in mitochondrial density in SPG11, although there was a trend for a decreased density (Figure 3B and Supplementary Figure 3A). For a specific analysis of the axonal compartment, microfluidic chamber slides were used to grow MNs and their axons in a polarized and homogeneous manner. Day 15 MN progenitors were seeded in microfluidic chamber slides. Up until 44 days, axon-like neurites had passed the grooves and extended into the axonal compartment (Figure 3C). We observed no significant changes in mitochondrial density in SPG11 axons (Figure 3D and Supplementary Figure 3B). Analysis of mitochondrial density in the soma also revealed no changes in SPG11 (Figure 3E and Supplementary Figure 3C).

As an impaired mitochondrial function within neurites may cause deficits in axonal transport (Kreiter et al., 2018), we compared the distribution of mitochondria between the proximal and distal parts of the microfluidic chamber grooves. The ratio of distal/total mitochondrial density was unchanged between controls and SPG11 (Figure 3F and Supplementary Figure 3D).

Using time-lapse microscopy, we additionally analyzed the transport of axonal mitochondria within microfluidic chambers (Figure 3G and Supplementary Videos 1, 2). In SPG11 MN axons, the ratio of moving mitochondria was severely reduced by 43% (Figure 3H and Supplementary Figure 3E). The ratio of anterogradely moving mitochondria was unchanged between groups, indicating that mitochondrial transport was affected bidirectionally (Figure 3I and Supplementary Figure 3F). Next, we analyzed the speed of moving mitochondria. We observed a significant reduction in the speed of axonal mitochondrial transport (Figure 3J and Supplementary Figure 3G). A subanalysis of the speed of antegradely moving mitochondria showed a significant reduction in SPG11 (Figure 3K and Supplementary Figure 3H). The subanalysis of retrograde axonal transport only showed a non-significant trend toward reduced retrograde mitochondrial transport speed in SPG11 MN (Figure 3L and Supplementary Figure 3I). This indicated that predominantly anterograde axonal mitochondrial transport is impaired in SPG11 MN.

In summary, analyses of the microfluidic chamber system showed that impaired axonal transport of mitochondria adds to the neuritic phenotype in SPG11 MNs whereas density of mitochondria was unchanged.

## Discussion

We aimed to investigate mitochondria in MNs derived from SPG11 patients with neuropathy. We also included MN derived from SPG11 knockout HUES6 and their isogenic control. Mitochondrial function was shown to be impaired in other types of complicated HSPs. Using a novel *in vitro* model of human iPSC derived MNs, we report neurite-specific alterations in SPG11. The observed defects include accumulation of electron-dense aggregates and lysosomes as well as functional and morphological mitochondrial alterations. Specifically, SPG11 mitochondria exhibited increased aspect ratio, reduced polarization, and reduced transport within axons.

### Overlapping Disease Mechanisms in SPG11 and SPG15

While pathogenic variants in other members of the AP5 complex, i.e., *ZFYVE26* (causing SPG15 HSP) and *AP5Z1* (causing SPG48 HSP), have recently been associated with mitochondriopathy (Denton et al., 2018), our data provide first human evidence that mitochondrial morphology and function is also altered in the more frequent SPG11 type of HSP. Expanding previous findings in cortical and dopaminergic neurons of SPG15 and SPG48, we show that mitochondrial transport is reduced in MNs of SPG11.

We describe a decrease in neuritic length of SPG11 MN, matching with the SPG11 phenotype in corticospinal MNs (Pérez-Brangulí et al., 2014). In SPG15 and SPG48 iPSC-derived neurons, decreased axonal length was reported in telencephalic MNs and dopaminergic neurons. Spinal MNs, however, were not analyzed (Denton et al., 2018). Both SPG15 and SPG48, are expressed in the spinal cord, arguing for a shared disease mechanism (Murmu et al., 2011; Pérez-Brangulí et al., 2014). For the present study, we selected SPG11 patients with a spastic paraplegia phenotype and severe neuropathy. Thus, it would be interesting to investigate whether mitochondrial alterations are also present in MNs derived from SPG11 patients without neuropathy.

Contrasting to the finding of decreased aspect ratio in SPG15 derived neurons (Denton et al., 2018), we here show data of a longer and thinner mitochondrial morphology in SPG11 MN. At the same time, membrane potential of mitochondria was also reduced in SPG11. Therefore, while we did not perform mechanistic studies, overlapping and differing disease mechanisms might be involved. Indeed, both defective mitochondrial fusion and fission have been proposed in MN models of genetic amyotrophic lateral sclerosis (Kodavati et al., 2020).

A detailed analysis of patients’ fibroblasts and HeLa cells had revealed that *SPG15* loss of function affected both formation and reformation of autophagosomes, whereas *SPG11* loss of function only affected the reformation step (Vantaggiato et al., 2019). Ultrastructurally, we observed electron-dense aggregates compatible with late autophagolysosomes within SPG11 MNs. These findings correlate with human sural nerve biopsy data (Hehr et al., 2007), human post-mortem spinal MN perikarya (Denora et al., 2016) and SPG11 knockout mouse cortical neurons and spinal neurons (Branchu et al., 2017). This confirms the validity of our model to recapitulate neuritic pathology in SPG11.

Mitochondrial dysfunction and impaired transport of mitochondria have been implicated in various neurodegenerative diseases including Charcot-Marie Tooth 2A and Parkinson’s (Kitada et al., 1998; Palacino et al., 2004; Lezi and Swerdlow, 2012; Stevens et al., 2015; Rizzo et al., 2016; Prots et al., 2018). Interestingly, increasing evidence supports a specific vulnerability of dopaminergic neurons in SPG11 (Paisan-Ruiz et al., 2010; Guidubaldi et al., 2011; Faber et al., 2018b; Pozner et al., 2020).

### Potential Underlying Mechanisms of Reduced Mitochondrial Transport

Mitochondrial impairment might be caused by the known lysosomal/autophagic dysfunction in SPG11 (Chang et al., 2014; Renvoisé et al., 2014; Vantaggiato et al., 2019; Darios et al., 2020; Khundadze et al., 2021), by alterations in mitochondrial trafficking, or by a direct function of mutant spatacsin on mitochondria. Interestingly, an extended biotechnological analysis, parts of which were released by (Huttlin et al., 2015), revealed interactions between spatacsin and FIS1^2^. FIS1 and DRP1 interact to promote mitochondrial fission (Serasinghe and Chipuk, 2016). This implicates a putative involvement of spatacsin in the mitochondrial fission pathway involving FIS1. Of note, it was hypothesized that a rescue of mitochondrial dynamics and autophagy may rescue axonal deficits in complicated types of HSP (Mou and Li, 2019).

We demonstrate that both the frequency and the velocity of axonal mitochondrial transport are impaired in SPG11 MNs. In light of previous evidence of impaired synaptic vesicle transport in SPG11, dysfunction of axonal transport may be a general feature of multisystem neuronal degeneration in SPG11 (Pérez-Brangulí et al., 2014). Correspondingly, axonal transport of mitochondria was significantly reduced in embryonic MNs in an SPG15 knockout mouse model (Khundadze et al., 2013). Lysotracker positive organelles and lysosomes were significantly increased in SPG11 MN, in accordance with previous studies showing an increased number of enlarged lysosomes in HeLa cells and human SPG11 fibroblasts (Chang et al., 2014; Renvoisé et al., 2014). Moreover, the presence of electron dense aggregates in SPG11 MN neurites matches similar findings in HeLa cells, iPSC derived corticospinal MNs and in both published SPG11 knockout mouse models (Renvoisé et al., 2014; Branchu et al., 2017; Khundadze et al., 2021). Interestingly, a peripheral nerve biopsy of the patient SPG11-2 had previously shown similar aggregates in the intermediate stage of this patient’s disease (Hehr et al., 2007).

Linking our findings of a reduced neurite growth in SPG11 MNs to previous studies that found abnormal axonal branching in SPG11 (Martin et al., 2012; Pérez-Brangulí et al., 2014), mitochondrial alterations in SPG11 might impair axonal maintenance and ultimately lead to the known phenotype of spastic paraplegia and motor neuropathy.

### Altered Mitochondrial Membrane Potential

Impaired mitochondrial membrane potential is an early sign of mitochondrial dysfunction and eventually cell death (Kodavati et al., 2020). Moreover, mitochondrial dysfunction has been demonstrated in different types of MN degeneration, including iPSC derived models of SPG15, CMT1A, and FUS (Saporta et al., 2015; Denton et al., 2018; Kreiter et al., 2018). For the analysis of mitochondrial membrane potential, no separation between the soma and neurite compartments was made. Consequently, we cannot conclude whether the observed changes are specific to neuritic mitochondria or affect the overall mitochondrial population.

### Limitations and Future Perspectives

While we provide multiple lines of evidence showing mitochondrial axonal dysfunction in SPG11 MN, a number of limitations have to be kept in mind when interpreting the data. Not all lines were used for specific experiments. Moreover, there was a considerable line to line variation (see Supplementary Figures 1–3), a well-known limitation when dealing with patient derived lines including iPSC and neural derivatives (Popp et al., 2018). Therefore, statistical analyses were conducted on the means of the respective lines. To compensate for the variability and to increase the number of cell lines, we included an SPG11 knockout embryonic stem cell line and its isogenic control line. In the future, it would be interesting to investigate mitochondrial morphology and function in additional neuronal populations and in more complex neuronal models, such as cerebral organoids.

## Conclusion

In summary, we established the differentiation of iPSC derived MNs from SPG11 patients with neuropathy and provided evidence of axonal mitochondrial alterations including altered mitochondrial morphology, polarization, and transport in SPG11 MNs. Analysis of mitochondria in other neuronal subtypes affected in SPG11 would help determine whether the observed mitochondrial phenotype is specific to MNs or directly linked to the dysfunction of spatacsin. Finally, therapeutic interventions targeting mitochondria may also rescue neuritic dysfunction and eventually neurodegeneration in SPG11.

## Data Availability Statement

The original contributions presented in the study are included in the article/Supplementary Material, further inquiries can be directed to the corresponding author.

## Ethics Statement

The studies involving human participants were reviewed and approved by the Ethikkommission der Friedrich-Alexander-Universität Erlangen-Nürnberg, Erlangen, Germany. The patients/participants provided their written informed consent to participate in this study.

## Author Contributions

BW and MR: conceptualization. FG, TP, FK, SL, IP, US-S, and MR: conduction of experiments, and analysis and interpretation of data. MR and JW: clinical care and data. FG and MR: manuscript initial draft. All authors contributed to manuscript critical correction and approval of final version.

## Conflict of Interest

The authors declare that the research was conducted in the absence of any commercial or financial relationships that could be construed as a potential conflict of interest.

## Footnotes

1 https://imagej.net/Coloc_2

2 https://thebiogrid.org/123177/summary/homo-sapiens/spg11.html

## Supplementary Material

Supplementary Figure 1 **(A)** Real-time quantitative PCR comparing day 0 (iPSC), day 20 (MN progenitors) and day 27 (mature MNs). Data are normalized to Control day 0. **(B)** Quantification of BrdU incorporation in d21 MN progenitors (*n* = 2 control and 2 SPG11 lines, no significant differences between Control and SPG11 means). Each dot represents BrdU ratio within one field of view. Per line, three fields of view were recorded from two technical replicates. **(C)** Quantification of Sox2 positive cells expressed as the ratio over DAPI positive cells in day 21 MN progenitors (*n* = 2 control and 2 SPG11 lines, no significant differences between Control and SPG11). Each dot represents Sox2 ratio within one field of view. Per line, three fields of view were recorded from two technical replicates. **(D)** Quantification of Hb9:GFP positive cells in MN as ratio over DAPI positive cells on day 32 (*n* = 3 control and 2 SPG11 lines, no significant differences between Control and SPG11). Each dot represents Hb9 ratio within one field of view. Per line, four fields of view were recorded from two technical replicates (Control-11: one replicate). **(E)** Black and white images from each color channel to clarify expression in Figure 1B. **(F)** Single data points for the analysis of total neurite length. Each dot represents neurite length of one Hb9 positive neuron, derived from two technical replicates per iPSC derived line and four technical replicates per HUES6 lines. **(G)** Single data points for MitoMorphology ImageJ plugin based quantification of mitochondrial morphology within MN somata. Each dot represents the mean of a defined region of interest of somata, derived from two technical replicates. **(H)** Single data points for manual tracing of mitochondrial outlines on EM images. Each dot represents one mitochondrion, derived from two cells per line and one technical replicate. **(I)** Single data points for MitoMorphology ImageJ plugin based quantification of mitochondrial morphology within MN axons. Each dot represents the mean of one axon; *n* = 2 technical replicates per line. **(J)** Single data points for manual tracing of mitochondrial outlines on EM images of neurites. Each dot represents one mitochondrion, derived from 10 to 15 neurites analyzed per line and one technical replicate.

Supplementary Figure 2 **(A)** JC-1 labeled MN were treated with CCCP (50 μM) for 30 min as a positive control, resulting in a loss of red JC-1 fluorescence. Single color channel magnified areas from boxes are shown on the right side of each panel. **(B)** Single data points for red/green quantification in JC-1 labeled cultures. Per line, six fields of view from three technical replicates (coverslips) were analyzed, resulting in a total number of mitochondria >100 per line. Boxes represent 25th – 75th percentiles and whiskers 5th – 95th percentiles. **(C)** Quantification of ultrastructural size of neuritic aggregates observed in SPG11 MNs, referring to Figure 2D. Each dot represents one aggregate, derived from 10 to 15 analyzed neurites per line and one technical replicate. **(D)** Single data points for the quantification of Lamp1 positive area. Each dot represents the Lamp1 positive area of 1 MN (*n* ≥ 10 per line), derived from two technical replicates. **(E)** Single data points of Lysotracker positive organelles in MN neurites. Each dot represents the density within one neurite (image width approximately 200 μm per neurite), *n* = 1 technical replicate per iPSC derived lines and two technical replicates per HUES6 derived lines. **(F)** Single channel micrographs of MitoDsRed/Lysotracker labeled neurites shown in Figure 2H. **(G,H)** Single data points for the manually quantified colocalizations of MitoDsRed labeled mitochondria and Lysotracker **(G)** and for Pearson’s R value. Each dot represents the density or Pearson’s R value within one neurite (image width approximately 200 μm per neurite), *n* = 2 technical replicates per line. Scale bar: **A**, 10 μm.

Supplementary Figure 3 **(A)** Quantification of mitochondrial density in non-polarized Mitotracker stained cultures for single lines, referring to Figure 3B. Each dot represents one neurite, derived from four fields of view and two technical replicates. **(B–D)** Quantification of mitochondrial density in axons **(B)**, in soma **(C)** and the ratio of proximal mitochondria over all mitochondria **(D)**, referring to Figures 3D–F, respectively. **(B)** Each dot represents density in one axon (image width approximately 200 μm per neurite), *n* = 2 technical replicates per line. **(C)** Each dot represents density in one soma, derived from two replicates. **(D)** Each dot represents one neurite, derived from one technical replicate. **(E–I)** Quantifications of axonal transport of mitochondria, shown for different lines, referring to Figures 3H–L, respectively. *n* = 2 technical replicates per line. **(E,F)** Each dot represents one neurite. **(G–I)** Each dot represents one mitochondrion.
