# Supplementary figures and images for: Axon-Specific Mitochondrial Pathology in SPG11 Alpha Motor Neurons
Source: Front Neurosci. 2021 Jul 7;15:680572. doi: 10.3389/fnins.2021.680572 (PMC8314181; doi:10.3389/fnins.2021.680572)

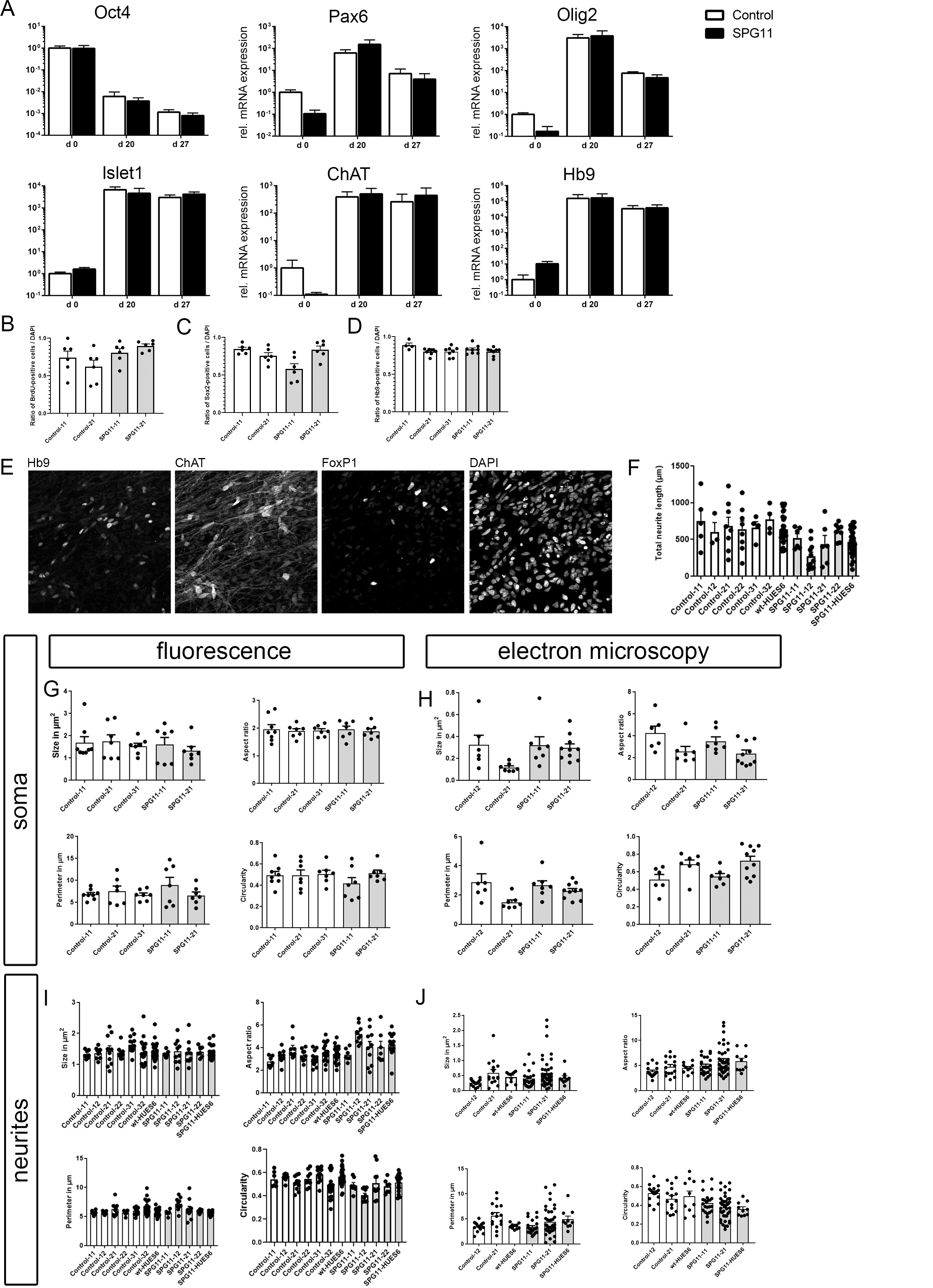

Supplement: Supplementary Figure 1 — (A) Real-time quantitative PCR comparing day 0 (iPSC), day 20 (MN progenitors) and day 27 (mature MNs). Data are normalized to Control day 0. (B) Quantification of BrdU incorporation in d21 MN progenitors (n = 2 control and 2 SPG11 lines, no significant differences between Control and SPG11 means). Each dot represents BrdU ratio within one field of view. Per line, three fields of view were recorded from two technical replicates. (C) Quantification of Sox2 positive cells expressed as the ratio over DAPI positive cells in day 21 MN progenitors (n = 2 control and 2 SPG11 lines, no significant differences between Control and SPG11). Each dot represents Sox2 ratio within one field of view. Per line, three fields of view were recorded from two technical replicates. (D) Quantification of Hb9:GFP positive cells in MN as ratio over DAPI positive cells on day 32 (n = 3 control and 2 SPG11 lines, no significant differences between Control and SPG11). Each dot represents Hb9 ratio within one field of view. Per line, four fields of view were recorded from two technical replicates (Control-11: one replicate). (E) Black and white images from each color channel to clarify expression in Figure 1B. (F) Single data points for the analysis of total neurite length. Each dot represents neurite length of one Hb9 positive neuron, derived from two technical replicates per iPSC derived line and four technical replicates per HUES6 lines. (G) Single data points for MitoMorphology ImageJ plugin based quantification of mitochondrial morphology within MN somata. Each dot represents the mean of a defined region of interest of somata, derived from two technical replicates. (H) Single data points for manual tracing of mitochondrial outlines on EM images. Each dot represents one mitochondrion, derived from two cells per line and one technical replicate. (I) Single data points for MitoMorphology ImageJ plugin based quantification of mitochondrial morphology within MN axons. Each dot represents the mea [file Image_1.TIF]

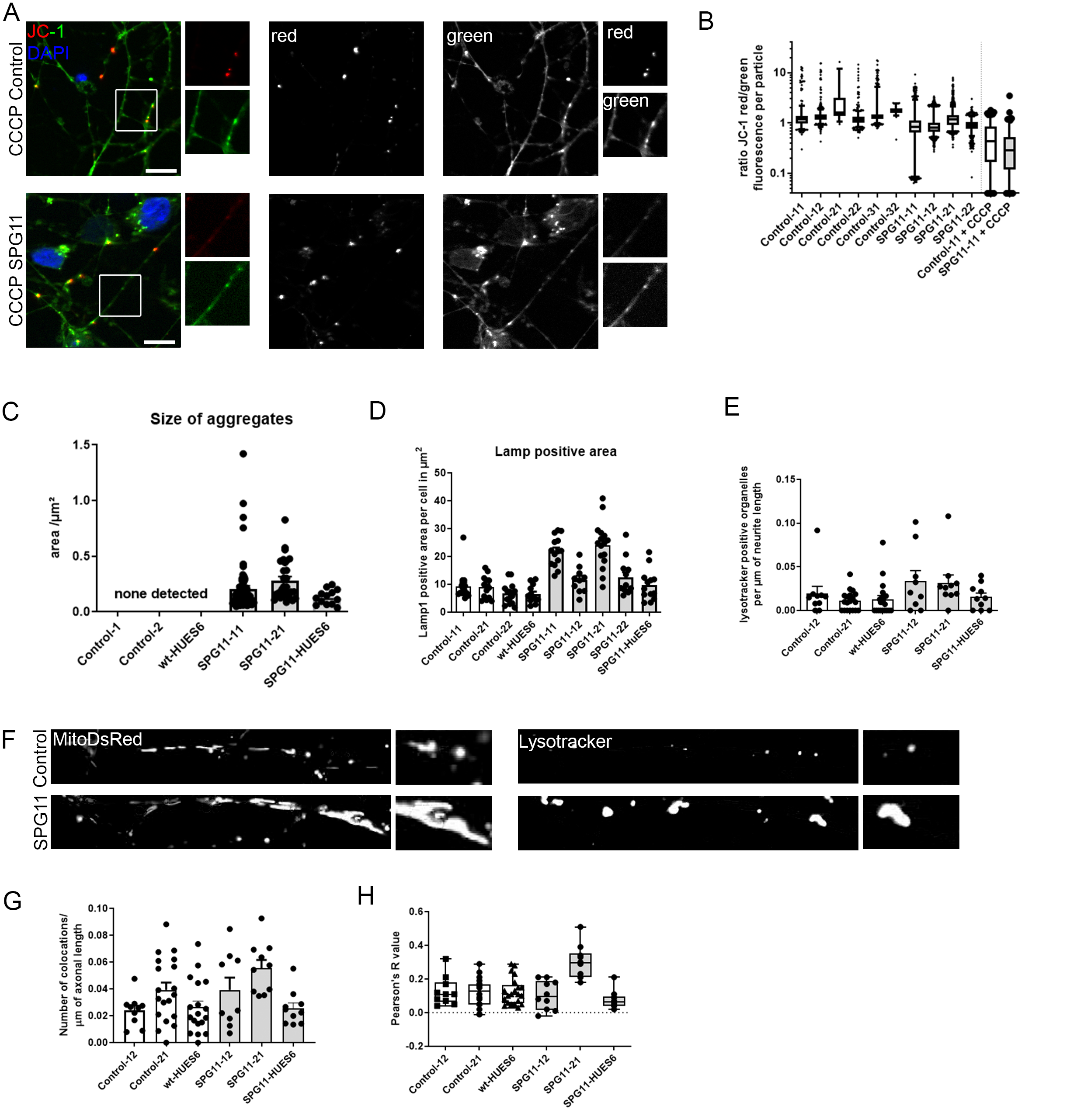

Supplement: Supplementary Figure 2 — (A) JC-1 labeled MN were treated with CCCP (50 μM) for 30 min as a positive control, resulting in a loss of red JC-1 fluorescence. Single color channel magnified areas from boxes are shown on the right side of each panel. (B) Single data points for red/green quantification in JC-1 labeled cultures. Per line, six fields of view from three technical replicates (coverslips) were analyzed, resulting in a total number of mitochondria >100 per line. Boxes represent 25th – 75th percentiles and whiskers 5th – 95th percentiles. (C) Quantification of ultrastructural size of neuritic aggregates observed in SPG11 MNs, referring to Figure 2D. Each dot represents one aggregate, derived from 10 to 15 analyzed neurites per line and one technical replicate. (D) Single data points for the quantification of Lamp1 positive area. Each dot represents the Lamp1 positive area of 1 MN (n ≥ 10 per line), derived from two technical replicates. (E) Single data points of Lysotracker positive organelles in MN neurites. Each dot represents the density within one neurite (image width approximately 200 μm per neurite), n = 1 technical replicate per iPSC derived lines and two technical replicates per HUES6 derived lines. (F) Single channel micrographs of MitoDsRed/Lysotracker labeled neurites shown in Figure 2H. (G,H) Single data points for the manually quantified colocalizations of MitoDsRed labeled mitochondria and Lysotracker (G) and for Pearson’s R value. Each dot represents the density or Pearson’s R value within one neurite (image width approximately 200 μm per neurite), n = 2 technical replicates per line. Scale bar: A, 10 μm. [file Image_2.TIF]

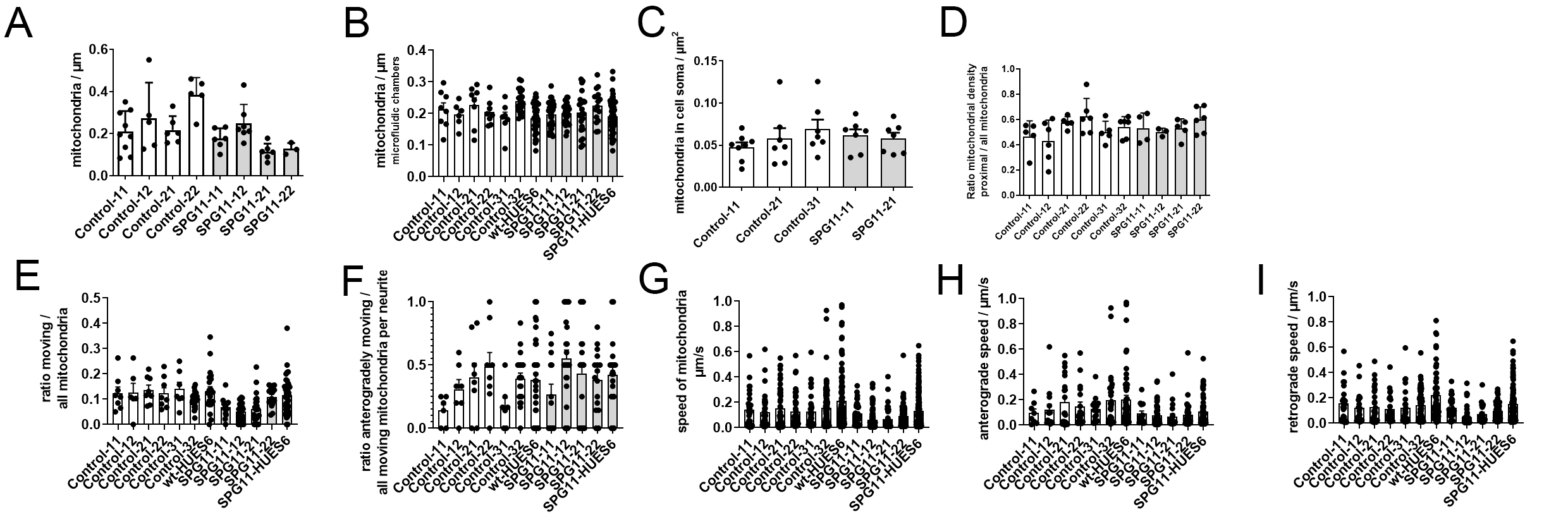

Supplement: Supplementary Figure 3 — (A) Quantification of mitochondrial density in non-polarized Mitotracker stained cultures for single lines, referring to Figure 3B. Each dot represents one neurite, derived from four fields of view and two technical replicates. (B–D) Quantification of mitochondrial density in axons (B), in soma (C) and the ratio of proximal mitochondria over all mitochondria (D), referring to Figures 3D–F, respectively. (B) Each dot represents density in one axon (image width approximately 200 μm per neurite), n = 2 technical replicates per line. (C) Each dot represents density in one soma, derived from two replicates. (D) Each dot represents one neurite, derived from one technical replicate. (E–I) Quantifications of axonal transport of mitochondria, shown for different lines, referring to Figures 3H–L, respectively. n = 2 technical replicates per line. (E,F) Each dot represents one neurite. (G–I) Each dot represents one mitochondrion. [file Image_3.TIF]
